# Nitric Oxide (NO) and Duchenne Muscular Dystrophy: NO Way to Go?

**DOI:** 10.3390/antiox9121268

**Published:** 2020-12-13

**Authors:** Cara A. Timpani, Kamel Mamchaoui, Gillian Butler-Browne, Emma Rybalka

**Affiliations:** 1 Institute for Health and Sport, Victoria University, Melbourne 8001, Victoria, Australia; emma.rybalka@vu.edu.au; 2 Australian Institute for Musculoskeletal Science, St Albans 3021, Victoria, Australia; 3 Institut de Myologie, Sorbonne University, INSERM UMRS974 Paris, France; kamel.mamchaoui@upmc.fr (K.M.); gillian.butler-browne@upmc.fr (G.B.-B.)

**Keywords:** Duchenne muscular dystrophy, metabolism, nitric oxide, nitrite, reactive oxygen species, superoxide

## Abstract

The discordance between pre-clinical success and clinical failure of treatment options for Duchenne Muscular Dystrophy (DMD) is significant. The termination of clinical trials investigating the phosphodiesterase inhibitors, sildenafil and tadalafil (which prolong the second messenger molecule of nitric oxide (NO) signaling), are prime examples of this. Both attenuated key dystrophic features in the *mdx* mouse model of DMD yet failed to modulate primary outcomes in clinical settings. We have previously attempted to modulate NO signaling via chronic nitrate supplementation of the *mdx* mouse but failed to demonstrate beneficial modulation of key dystrophic features (i.e., metabolism). Instead, we observed increased muscle damage and nitrosative stress which exacerbated MD. Here, we highlight that acute nitrite treatment of human DMD myoblasts is also detrimental and suggest strategies for moving forward with NO replacement therapy in DMD.

## 1. Introduction

The failure of therapeutic candidates to translate from pre-clinical animal work to clinical trial success is unfortunately a common occurrence. This is particularly true for the muscle wasting condition, Duchenne Muscular Dystrophy (DMD), a rare and incurable disease that is fatal in all cases. Gene and exon skipping therapies, which partially restore the ablated dystrophin protein back into the muscle membrane, are the only curative options. However, therapeutic use is limited to those with specific dystrophin gene mutations (for exon skipping) and long-term safety and efficacy is yet to be established (for both therapies). Thus, new treatment options are crucial, particularly since standard-of-care corticosteroid practice is associated with side-effects which limits clinical utility. 

## 2. Main

Re-purposing drugs is of particular benefit for rare diseases such as DMD as it can expedite new therapeutic options since it leverages off previous safety and tolerability trials in other diseases. One example of re-purposed drugs for the treatment of DMD are the phosphodiesterase (PDE) type 5 inhibitors, sildenafil and tadalafil, both commonly prescribed for erectile dysfunction [1]. PDE inhibition enhances blood flow by increasing the half-life of cyclic guanosine 3′,5′-monophosphate (cGMP), the second messenger of nitric oxide (NO) [1]. Pre-clinically, these PDE5 inhibitors prevented skeletal muscle fatigue, ischemia and damage in the dystrophin-deficient *mdx* mouse indicating that manipulation of the NO pathway is beneficial to dystrophic muscles [2,3,4,5,6,7]. Clinically, however, both have failed to modify primary endpoint measures, being delayed loss of ambulation (tadalafil; NCT01865084) [8] and improved cardiac function (sildenafil; NCT01168908) [9] in DMD patients, resulting in the termination of both trials. Similarly, sildenafil had no effect in Becker Muscular Dystrophy patients [9,10]. While there are no individualized data reported for the tadalafil trial (NCT01865084), there was evidence of participant-specific responsivity in the sildenafil trial where 4/14 participants (29%) experienced worsening of cardiac function with sildenafil versus 1/8 (12.9%) on placebo and the other 10/14 showed no response [9]. These data suggest that other factors interplay to orchestrate the biological effects of enhanced NO signaling in DMD.

In skeletal muscle, NO, produced by neuronal nitric oxide synthase (nNOS), is a critical signaling molecule that modulates key physiological processes including blood flow, contraction, glucose uptake and metabolism, among others [7]. In dystrophic muscles, nNOS dissociates from the sarcolemma resulting in its targeted degradation [11,12,13] and consequently, a diminished capacity for NO generation [7,14,15]. Reduced NO bioavailability impairs many processes including glucose metabolism [16]. This contributes to the global metabolic dysfunction (and reduced adenosine triphosphate (ATP) production) observed in dystrophic muscles [17,18,19], which we postulate is a key driver of dystrophic pathology since metabolic efficiency is essential for cell life [20]. This led us to question whether normalizing NO levels could have therapeutic potential. Since stimulating nNOS (i.e., with l-arginine supplementation [21,22]) to enhance endogenous NO generation is limited by the lower native nNOS protein content in dystrophic muscles, we sought to increase NO availability through nNOS-independent means. The nitrate-nitrite-NO pathway is an nNOS-independent mechanism that expands the NO pool [23]. Commensal bacteria reduce nitrate to nitrite (NIT), which is enzymatically converted in the blood/tissues to NO [23]. Nitrate supplementation improves mitochondrial efficiency and ATP production in healthy individuals [24], key parameters, which if improved, should theoretically mitigate the dystrophic condition. 

In contrast to our hypothesis, in *Timpani* et al. we demonstrated that 8-week long treatment of dystrophic *mdx* mice with 1 mM nitrate was deleterious rather than therapeutic [25]. Despite nitrate increasing citrate synthase activity (a marker of mitochondrial density), it exacerbated mitochondrial uncoupling, muscle damage and nitrotyrosine staining (peroxynitrite marker) in dystrophic muscle [25]. The reaction of NO with superoxide results in the highly reactive nitrogen species, peroxynitrite, which damages proteins. While nitrate supplementation also increased peroxynitrite content in healthy control muscles (82% vs. 2775% increase in untreated vs. nitrate supplemented muscles, respectively), there was no evidence of concomitant muscle damage as was seen in dystrophic muscles [25]. This strain-dependent effect reflects the well-documented oxidative stress in dystrophic *mdx* muscles [26,27] and highlights that uncontrolled NO generation within a pro-oxidant environment exacerbates muscle damage. It is interesting to speculate whether variations in dietary antioxidant intake or endogenous antioxidant responsivity could explain why some patients experienced worsening of cardiomyopathy while others did not in the sildenafil trial [9].

We postulated that the detrimental effect of nitrate supplementation in the *mdx* mouse might be due to the chronic treatment period used. To test this hypothesis, here we cultured immortalized human DMD (fascia-lata of a 10 year old male with an exon 52 deletion in the dystrophin gene) and healthy control (CON; paraspinal muscle of a 12 year old female) myoblasts as described previously by us [28] (supplementary information) with 1 mM NIT (nitrate cannot be converted to NIT in cell models) for 24 h. Cells were grown in medium containing low glucose 199 media (1:5, Gibco 11150059; purchased from ThermoFisher Scientific (Scoresby, Australia)), high glucose Dulbecco’s modified eagle medium (DMEM; 4:5, Gibco 10566016), fetuin (25 µg/mL, Sigma Aldrich (Sydney, Australia) F2379), human epidermal growth factor (5 ng/mL; Sigma Aldrich E9644), basic human fibroblast growth factor (0.5 ng/mL, Sigma Aldrich F0291), insulin (5 µg/mL, Sigma Aldrich I5500), dexamethasone (0.2 µg/mL, Sigma Aldrich D4902), gentamycin (50 µg/mL, Gibco 15750060) and fetal bovine serum (20%, Bovogen Biologicals (East Keilor, Australia)). Cells were seeded at a confluency of ~10% in 75 cm^2^ flasks and passaged every 3 days and experiments were performed on undifferentiated myoblasts. Following NIT treatment, mitochondrial viability was determined as described previously [29] using 5000 cells per well and a positive control of FCCP and antimycin A (3 µM each) to dissipate the inner mitochondrial membrane potential (ΔΨ). Mitochondrial respiratory function (including spare respiratory capacity, ATP production and coupling efficiency) and extracellular acidification rate (ECAR), a measure of anaerobic glycolysis, were determined using the Seahorse Bioscience XF24 Analyzer as described in [28] with some modifications. On gelatin-coated (0.5%) Seahorse XF24 cell culture V7 microplates, CON (25,000 cells per well) and DMD myoblasts (15,000 cells per well) were seeded and treated with NIT. 24 h later, media was replaced with pre-warmed assay buffer (DMEM XF assay media (Seahorse Bioscience (North Billerica, MA, USA), 102365-10), 25 mM glucose and 1 mM sodium pyruvate; pH 7.4). To a pre-hydrated XF24 Sensor Cartridge, 50 µL of oligomycin (final concentration of 3 µM), 55 µL of FCCP (final concentration of 3 µM) and 60 µL of antimycin A and rotenone (final assay concentration of 1 µM and 2 µM, respectively) was added to ports A, B and C respectively. Finally, mitochondrial superoxide production was measured as described in [28] using antimycin A (3 µM) as a positive control to induce superoxide generation. Two-way ANOVA was used to detect between genotype and treatment differences. When a main effect/interaction was detected, unpaired T-tests were used to identify between group differences using SPSS (version 21; Armonk, NY, USA). An α value of 0.05 was considered significant. 

**Figure 1:**
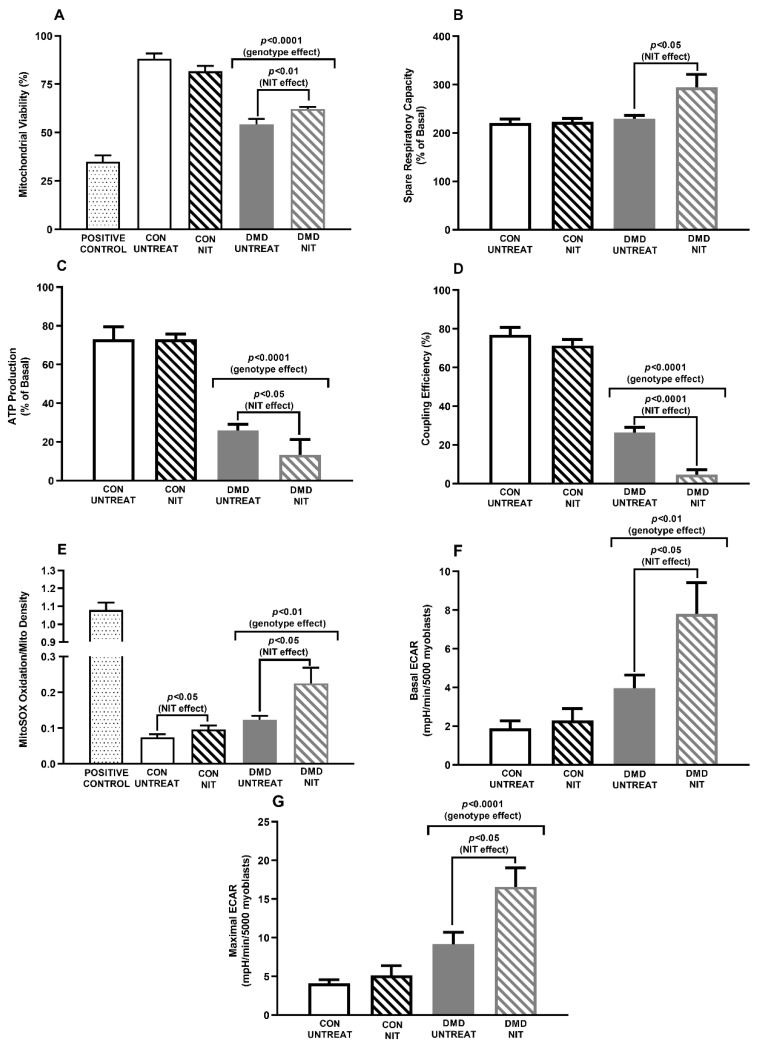
Mitochondrial viability, functional indices and superoxide production and extracellular acidification rate (ECAR) of untreated (UNTREAT) and nitrite (NIT) treated control (CON) and Duchenne Muscular Dystrophy (DMD) myoblasts. Cells were treated for 24 h with 1 mM NIT. Mitochondrial viability was reduced in DMD myoblasts compared to CON (*p* < 0.0001), but NIT supplementation increased this parameter in DMD myoblasts (*p* < 0.01; **A**). Spare respiratory capacity, adenosine triphosphate (ATP) production and coupling efficiency (mitochondrial functional indices) were determined using the Seahorse Bioscience XF24 Analyzer with data presented per 5000 myoblasts due to different plating densities of CON and DMD myoblasts. While NIT increased the spare respiratory capacity in DMD myoblasts (*p* < 0.05; **B**), NIT further reduced ATP production (*p* < 0.05; **C**) and coupling efficiency (*p* < 0.0001; **D**) in DMD myoblasts. ATP production and coupling efficiency are already reduced in DMD myoblasts compared to CON (*p* < 0.0001). Mitochondrial superoxide production was higher in DMD myoblasts compared to CON (*p* < 0.01; **E**) and NIT treatment increased it in both CON and DMD myoblasts (*p* < 0.05). Extracellular acidification rate (ECAR), a metric of glycolytic flux, was measured using the Seahorse Bioscience XF24 Analyzer concomitantly with mitochondrial function. Basal ECAR (**F**) and maximal ECAR (**G**) were significantly higher in DMD myoblasts compared to CON (*p* < 0.01 and *p* < 0.0001 respectively) and NIT further stimulated ECAR in DMD myoblasts only (*p* < 0.05). Results are presented as mean ± standard error of the mean. Mitochondrial viability and superoxide assays: *n* = 4 per group; Seahorse assays: *n* = 7–8 CON UNTREAT, *n* = 6 CON NIT, *n* = 5 DMD UNTREAT, *n* = 5 DMD NIT.

Despite acute NIT treatment increasing mitochondrial viability (*p* < 0.01 DMD NIT vs. DMD untreated (UNTREAT); Figure 1A) and spare respiratory capacity (*p* < 0.05 DMD NIT vs. DMD UNTREAT; Figure 1B), this did not translate to improvements in other key metabolic parameters. In fact, acute NIT treatment exacerbated the repression of ATP production (*p* < 0.05 DMD NIT vs. DMD UNTREAT; Figure 1C) and mitochondrial uncoupling (*p* < 0.001 DMD NIT vs. DMD UNSUPP; Figure 1D) already evident in DMD myoblasts. While mitochondrial uncoupling could be considered protective against oxidative stress, in DMD myoblasts it only caused severe impact to ATP synthesis without any attenuation of mitochondrial superoxide, which was 83% higher in NIT-treated compared to UNTREAT dystrophic myoblasts (*p* < 0.05 DMD NIT vs. DMD UNTREAT; Figure 1E). Interestingly, though, mitochondrial superoxide content decreased with extended NIT treatment of 3 and 7 days in DMD myoblasts (by 66% and 93%, respectively compared to levels detected at 24 h; data not shown). Ostensibly, these data suggested to us induction of the endogenous antioxidant response to oxidative stress sufficient to attenuate detectable superoxide, which is a time-dependent event. However, when these data are considered in context of our data from nitrate-treated *mdx* mice, the attenuation of detectable superoxide seems more likely due to the vigorous formation of peroxynitrite. Our data demonstrate that amplifying NO bioavailability in an acute manner worsens the metabolic crisis in DMD cells and drives oxidative and nitrosative stress.

Augmentation of the spare respiratory capacity reflects the known metabolic modulatory effects of NO on glycolytic flux, observed here through NIT-stimulated ECAR (*p* < 0.05 DMD NIT vs. DMD UNTREAT; Figure 1F,G). Activity of phosphofructokinase (PFK), the rate-limiting enzyme of glycolysis, is reduced in DMD patients [30] which appears to be caused through loss of allosteric control by nNOS/NO [16]. Our observation suggests that in DMD myoblasts, PFK is activated by NIT-derived NO, which stimulates glycolytic flux and pyruvate shuttling into the mitochondria for the stimulation and expansion of the mitochondria tricarboxylic acid (TCA/Krebs) cycle. The outcome is a higher proton gradient across the inner mitochondrial membrane i.e., increased spare respiratory capacity. Paradoxically, this did not result in greater ATP production but rather in proton leak (most likely through uncoupling proteins) which consumes the proton gradient without ATP yield. Thus, the NO-driven compensatory stimulation of glycolytic flux cannot overcome the mitochondrial dysfunction to re-balance energy homeostasis in DMD myoblasts.

**Figure 2:**
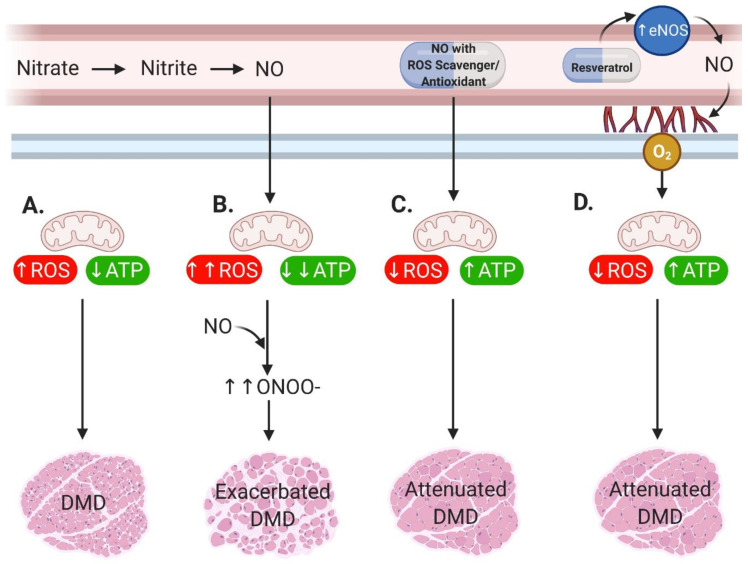
Proposed approach for nitric oxide (NO) therapy in Duchenne Muscular Dystrophy (DMD). (**A**) Dystrophic muscle is characterized by mitochondria which produce elevated reactive oxygen species (ROS) and reduced adenosine triphosphate (ATP). Along with other pathological processes, this culminates in muscle damage and wasting which impairs function. (**B**) We have previously demonstrated that the use of chronic nitrate supplementation to increase the NO pool is detrimental in dystrophic murine muscle. The superfluous ROS reacts with the NO to produce peroxynitrite (ONOO^–^) which amplifies muscle damage. As such, manipulation of the nitrate-nitrite-NO pathway and NO donor/signal amplifier drugs are unfeasible as a treatment for DMD as they currently stand. (**C**) Since we observed that the increased muscle damage in dystrophic murine muscle was accompanied by markers of higher regenerative capacity (i.e., centronucleated muscle fibers), we postulate that a combination therapy/dual function small molecule which increases NO bioavailability while addressing the oxidative stress may be beneficial rather than damaging. (**D**) Alternatively, increasing endogenous NO signaling e.g., through endothelial NOS (eNOS) with compounds such as resveratrol could circumvent the loss of nNOS-mediated NO signaling and restore physiologically relevant NO levels to induce benefits such as enhanced capillarization and muscle oxygenation. Image created with BioRender.com.

While our research suggests that manipulation of NO (particularly by the nitrate-NIT-NO pathway) is pernicious in dystrophic muscles, there does appear to be some merit in pursuing this treatment avenue. We saw more centronucleated fibers in nitrate-treated dystrophic muscles which suggests augmentation of muscle regeneration [25]. It stands to reason that if we could exploit this beneficial aspect of NO donor therapy while concomitantly buffering superoxide to prevent the oxidative/nitrosative damage elicited, then the true capacity for NO donor therapeutics would be known. For example, when sildenafil is administered in conjunction with vitamin E (a known free oxygen radical scavenger) in diabetic rats, vitamin E enhances the therapeutic effect of sildenafil [31]. If such a molecule were available, it could restore mitochondrial ATP production and energy homeostasis to lessen damage in the first place, but also facilitate regeneration to halt disease progression. Alternatively, other endogenous NO-generating enzymes such as endothelial NOS (eNOS) could be exploited to circumvent the loss of nNOS-mediated NO signaling and naturally elevate NO signaling within the vicinity of skeletal muscle tissue at beneficial physiological concentrations. For example, resveratrol treatment has been shown to increase eNOS expression [32] and skeletal muscle capillarization and oxygenation through eNOS-dependent NO signaling [33], and in *mdx* mice this leads to improved endurance [34] (Figure 2).

## 3. Conclusions

Here, we present the contraindicative nature of enhancing NO bioavailability as a treatment for DMD. Our previous findings in nitrate-treated *mdx* mice indicate that chronic NO (via nitrate) supplementation promotes nitrosative stress and muscle damage while here, acute NO (via NIT) supplementation of immortalized myoblasts derived from a DMD patient amplifies metabolic perturbations (with a flow on escalation of nitrosative stress likely). Taken with the failed clinical trials using PDE5 inhibitors to prolong NO signaling, it seems that clinical success through NO manipulation is unlikely. However, rather than abandon NO as a treatment option for DMD, modifying our approach may be the key. We suggest that a combination therapy or dual function small molecule, which increases the NO pool while addressing the oxidative stress (i.e., antioxidative capacity), may prove successful for the treatment of DMD (Figure 2). This should be tested in a human-based cell culture system (such as immortalized myoblasts/tubes derived from patients) in the first instance, which may improve translational outcomes over testing only in non-genetically and/or -phenotypically homologous animal models.
